# Personalization of Words in Anomia Treatment for People With Aphasia: A Scoping Review

**DOI:** 10.1044/2026_AJSLP-25-00512

**Published:** 2026-05-08

**Authors:** Amy Vogel-Eyny, Mara Steinberg Lowe, Sameer Ashaie, Samantha Slattery, Brooke Boxrud, Nichol Castro

**Affiliations:** a Department of Speech-Language Pathology and Audiology, Hunter College, New York, NY; b Department of Linguistics and Communication Disorders, Queens College, New York, NY; c Department of Communication Sciences and Disorders, Northwestern University, Evanston, IL; d Department of Physical Medicine and Rehabilitation, Feinberg School of Medicine, Northwestern University, Chicago, IL; e Think and Speak Lab, Shirley Ryan AbilityLab, Chicago, IL; f Hunter College Libraries, CUNY Hunter College, New York, NY; g Department of Communicative Disorders and Sciences, University at Buffalo, NY

## Abstract

**Purpose:**

This scoping review examined how “personalization” of treatment targets, specifically words, has been defined and implemented in anomia interventions for people with aphasia following stroke or primary progressive aphasia. The review identified (a) how studies define personalized words, (b) the methods used to select them, and (c) the linguistic characteristics of personalized targets.

**Method:**

Following the Preferred Reporting Items for Systematic Reviews and Meta-Analyses Extension for Scoping Reviews, systematic searches were conducted in MEDLINE, PsycINFO, Web of Science, and CINAHL on July 8, 2024, with no date limits. Eligible studies reported treatment involving personalized single-word targets for adults with aphasia. Data extraction captured how personalization was defined or described, word selection methods, involvement of participants and partners, and linguistic characteristics. Descriptor terms were analyzed for semantic relatedness using the WordNet LESK algorithm to identify higher order dimensions of the concept “personalization.”

**Results:**

Twenty-one studies met inclusion criteria. Few provided explicit definitions; most described personalization using descriptors such as “relevant,” “functional,” “familiar,” “useful,” “frequent,” and “meaningful.” Network analysis grouped these into three dimensions: meaningful, functional, and frequent. Common selection methods included interviews (*n* = 11), free listing (*n* = 8), performance informed (*n* = 7), and category based (*n* = 5). All studies involved participants with aphasia; most (*n* = 14) included a communication partner.

**Conclusions:**

Personalization is widely referenced in anomia treatment but rarely operationalized. Clearer reporting of personalization dimensions, selection methods, and linguistic features would improve comparability and align research with person-centered frameworks such as the Life Participation Approach to Aphasia and the International Classification of Functioning, Disability and Health.

**Supplemental Material:**

https://doi.org/10.23641/asha.32118373

Aphasia is a language disorder caused by acquired brain damage, resulting in difficulties with understanding, producing, and processing language across various modalities (Palmer et al., 2017). One of the most common, persistent, and debilitating symptoms is word retrieval difficulty, known as “anomia,” which can make communication effortful and frustrating (Cordella et al., 2024; Larfeuil & Dorze, 1997; Reilly et al., 2011). Anomia most often arises following stroke; however, it is also an early and prominent impairment symptom of primary progressive aphasia (PPA), a neurodegenerative disorder (Flurie et al., 2020). Poststroke aphasia is particularly prevalent, affecting approximately one third of stroke survivors, with an estimated 2.5 million individuals in the United States currently living with poststroke aphasia (Brady et al., 2012; Simmons-Mackie & Cherney, 2018). In contrast, PPA is much rarer, with an estimated 50,000 individuals living with the condition in the United States (National Institutes of Health, n.d.).

Speech and language therapy (SLT) is the primary intervention for managing anomia in poststroke aphasia, aiming to restore language and improve functional communication by supporting word retrieval and other language processes (Fridriksson & Hillis, 2021). SLT is similarly used in managing word-finding difficulties in progressive aphasia; however, treatment goals often shift toward compensatory strategies and maintaining communication over time as the condition advances (Flurie et al., 2020; Robinaugh & Henry, 2022). Numerous interventions have been developed and support the efficacy of anomia treatment in poststroke aphasia (Nickels, 2002; Raymer & Roitsch, 2023). In more recent years, research on word-finding interventions in PPA has expanded, with emerging evidence indicating similarly promising outcomes (Cadório et al., 2017; Jokel et al., 2014; Robinaugh & Henry, 2022). Together, this work reflects a large and well-developed body of empirical and theoretical research on anomia treatment, providing a strong foundation for examining how personalization of lexical targets has been conceptualized within aphasia.

Nonetheless, a persistent challenge in both poststroke aphasia and PPA interventions is the limited transfer of treatment effects beyond the specific words targeted during therapy, also called “generalization” (Cadório et al., 2017; Mayer et al., 2024; Webster et al., 2015). This raises important questions about how therapy materials are selected (Mason et al., 2011). When gains are narrowly confined to practiced items, the communicative value of therapy may depend less on the number of words trained and more on their personal and contextual relevance to the individual (Robinaugh & Henry, 2022). Unlike interventions that target decontextualized word lists, interventions that prioritize words aligned with a person's daily routines, social relationships, and personal identity may better support real-life communication (Renvall et al., 2013a, 2013b). While research on other interventions that target functional communication for people with aphasia, such as alternative and augmentative communication, has highlighted the importance of tailoring vocabulary to an individual's demographic and social context (e.g., age, gender, roles, and interests; Dietz et al., 2013; Thiessen & Brown, 2021), this approach has only recently begun to inform word selection in anomia treatment (Renvall et al., 2013b, 2013a). Given the challenge of limited generalization, a more deliberate focus on aligning words for therapy with each individual's communicative priorities is crucial to achieving meaningful functional outcomes.

Evidence that directly tests the impact of personalization on anomia treatment outcomes remains limited. Most prior work showed advantages for personalized targets that are trained relative to an untrained set of generic targets (e.g., Flurie et al., 2020; Kristensson et al., 2022). Cherney et al. (2015), one of the few studies to evaluate the effect of personalization during therapy, found larger, clinically meaningful gains for “personally relevant” items than for generic items embedded in the same scripts among individuals with poststroke aphasia. These findings suggest potential benefits of personalization, but they do not establish how personalization should be defined or operationalized. For this reason, clearer definition and reporting of how personalization is implemented are needed before its effects on anomia treatment outcomes can be meaningfully evaluated.

Theoretical work supports using individualized target words for people experiencing anomia. Raymer et al. (2008, p. S266) define salience as “the perceived value or relevance of the experience to the participant,” highlighting the role of subjective importance in shaping therapeutic engagement. This definition aligns with the saliency principle of neuroplasticity, which suggests that experiences perceived as meaningful or motivating are more likely to activate neural systems involved in attention, memory, and learning—mechanisms that support experience-dependent plasticity (Kleim & Jones, 2008; Raymer et al., 2008). This emphasis on personal relevance is also consistent with the Rehabilitation Treatment Specification System, which identifies volitional ingredients—including motivation, engagement, and goal relevance—as active components of rehabilitation interventions that can influence treatment outcomes (e.g., Van Stan et al., 2019). In aphasia rehabilitation, this principle has been cited to justify selecting therapy targets that reflect a person's identity, routines, or goals. However, as Renvall et al. (2013a) pointed out, salience has been used inconsistently in the aphasia literature and often serves as a loosely defined proxy for concepts such as meaningfulness or functional relevance. Moreover, despite frequent theoretical claims that personal relevance enhances salience and drives neuroplastic change, there are, to our knowledge, no mechanistic studies that have directly tested this assumption. At present, the proposed relationship between personal relevance, salience, and neural plasticity remains a theoretical prediction rather than an empirically verified mechanism.

Complementing this neurobiological perspective, the Life Participation Approach to Aphasia (LPAA) offers a client-centered philosophy that positions personal goals, values, and meaningful participation at the forefront of treatment planning. LPAA encourages clinicians to focus not only on language performance but also on enabling reengagement in daily life activities that matter most to individuals with aphasia. Similarly, the World Health Organization International Classification of Functioning, Disability and Health provides a holistic framework that situates impairments within the broader context of personal and environmental factors, emphasizing activity and participation as critical domains of functioning (World Health Organization, n.d.). Together, these models reinforce the clinical and ethical rationale for personalizing therapy.

## This Study

Given challenges in generalization, a more deliberate focus on aligning word targets with each individual's communicative priorities may be crucial to achieving meaningful functional outcomes. At the same time, practical barriers—such as high caseloads and limited therapy time—can make it difficult to fully individualize treatment (Thiessen & Brown, 2021). However, challenges related to personalization extend beyond time burden alone: The concept is inconsistently defined, operationalized, and reported across studies, limiting comparability and complicating efforts to translate personalization into routine practice. These constraints reinforce the need for greater clarity around what “personal relevance” means and how it can be implemented in clinical and research settings. Some authors invoke theoretical frameworks such as the saliency principle of neuroplasticity, while others emphasize person-centered care, autonomy, or general engagement. Still, many studies reference personalization without explicitly defining it or detailing how it informs word selection.

The present study examines how personalization of treatment words is represented in the anomia treatment literature for poststroke aphasia and PPA. Specifically, this scoping review addresses the following questions:

1. How do previous studies define “personalized” words?
2. How do studies obtain or select these words?
3. What types of personalized words are used in anomia treatment?

## Method

Our goal in this review was to chart how personalization of treatment words has been conceptualized and applied across studies of anomia treatment in people with aphasia. Because the purpose was to describe the extent and variation in this body of work, a scoping review methodology was selected. In line with the Preferred Reporting Items for Systematic Reviews and Meta-Analyses Extension for Scoping Reviews (PRISMA-ScR) guidelines (Tricco et al., 2018), studies were not subjected to formal quality appraisal. This project did not have a registered protocol.

### Study Identification

Study identification and retrieval were conducted in accordance with the PRISMA-ScR guidelines (Tricco et al., 2018) and the *JBI Manual for Evidence Synthesis* guidance for scoping review search strategies (Peters et al., 2020). A comprehensive search strategy, which employed both subject headings and key words, was run in MEDLINE (Ovid), PsycINFO (EBSCO), Web of Science Core Collection, and CINAHL (EBSCO) on July 8, 2024, with no date restrictions. The search queries were designed to yield only full-length, peer-reviewed articles in relevant fields and to exclude conference proceedings. The full-search queries were developed in consultation with a librarian (S.S.) and are available in the Appendix. Search results were exported and de-duplicated into the Covidence software (Veritas Health Innovation, n.d.)

### Eligibility Criteria and Study Selection

Articles were eligible for inclusion if they reported original treatment data targeting anomia in adults with poststroke or progressive aphasia. Studies were required to include personalized single words within the intervention, be published in English, and appear in peer-reviewed journals. Exclusion criteria were studies involving adults with other etiologies (e.g., traumatic brain injury or neoplastic causes), dementias other than PPA, interventions not addressing anomia, or publications in the form of systematic reviews, meta-analyses, conference abstracts, theses, or other unpublished literature.

After de-duplication, the search yielded 864 articles. Four reviewers (A.V.-E., M.S.L., N.C., and B.B.) independently screened titles and abstracts against the inclusion and exclusion criteria. To ensure reliability between reviewers, a series of training exercises was conducted prior to screening, including a pilot test on a random sample of 20 citations. The full texts of articles that passed title and abstract screening were then independently reviewed by the same four reviewers, with two “yes” votes required for inclusion at both the title/abstract and full-text stages and any conflicts at either stage resolved through discussion among all four reviewers in consultation with S.A. A PRISMA flow chart of study identification with reasons for exclusion is provided in Figure 1. During data extraction, we identified two studies that had passed full-text eligibility but defined “personalization” in an unanticipated way—solely on the basis of participant performance (e.g., items added to the training set if named incorrectly at baseline). Because our review focused on personalization defined in terms of personal relevance rather than performance accuracy, these studies were excluded at the extraction stage. In addition, we recognized that our database strategy alone was insufficient to identify all relevant studies, as several eligible articles did not reference personalization or related concepts in their titles or abstracts, despite the inclusion of multiple personalization-related search terms (see the Appendix). To address this limitation, we conducted supplementary reverse citation chaining in accordance with the *JBI Manual* recommendations for scoping review searches (Peters et al., 2020), systematically reviewing the reference lists of included papers as well as additional relevant articles known to the authors. This process yielded 556 additional articles for full-text review. Two authors (A.V.-E. and M.S.L.) independently reviewed each article, with inclusion determined through consensus, resulting in 12 additional eligible studies. In total, 21 studies met inclusion criteria and were included in the final review. It is important to note that the studies identified through reverse citation chaining contained no abstract-level reference to personalization or related concepts and therefore could not have been captured through additional or alternative search terms.

**Figure 1.**
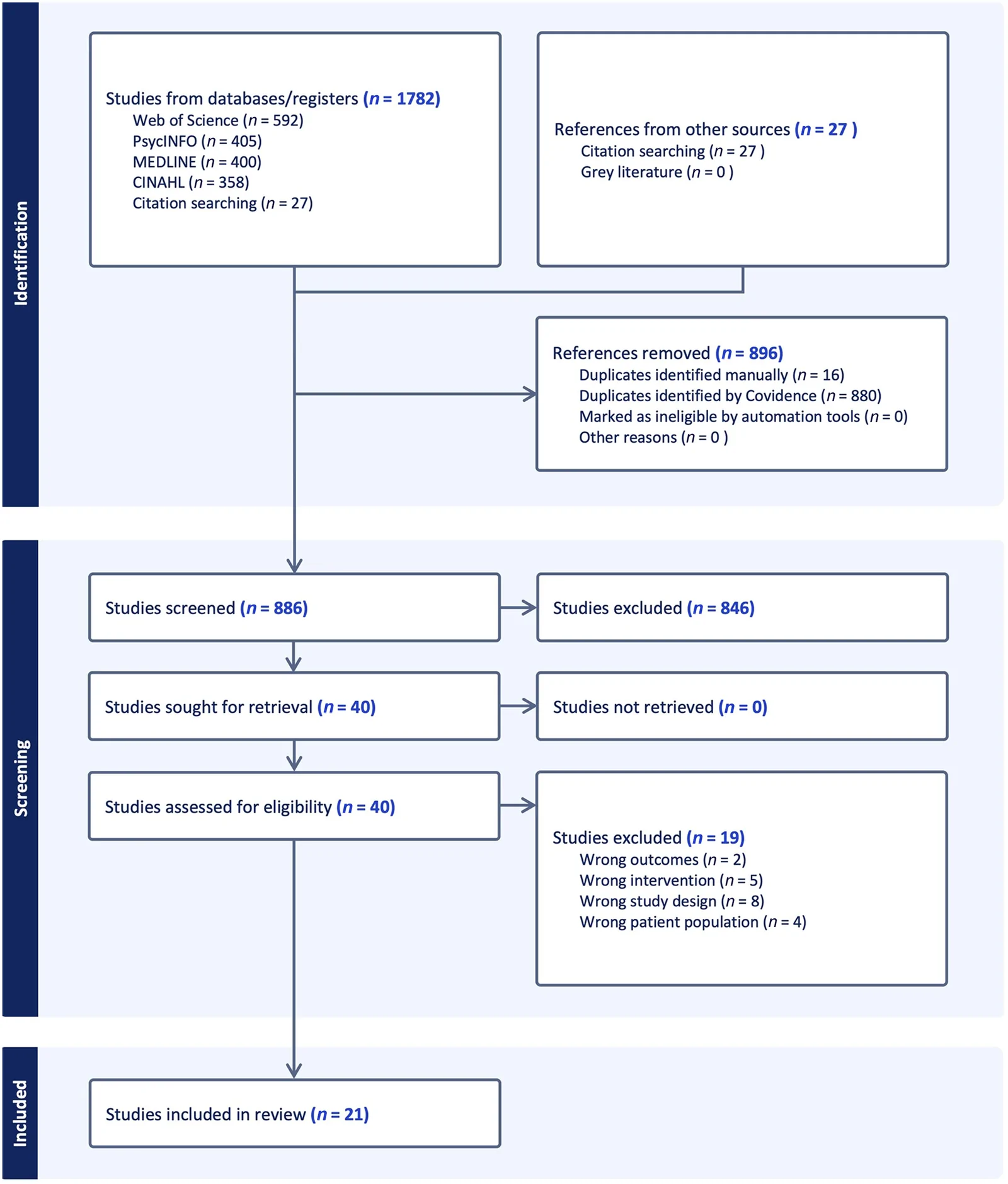
Screening of studies.

### Data Extraction and Synthesis

Bibliographic details were collected from each included study (author, year of publication, diagnosis, sample size), along with study characteristics most relevant to the review questions. Information was extracted on how personalization was defined, the strategies used to select personalized items (e.g., free listing, interview/conversation based, script based, category based, performance based), and the extent to which participants or communication partners were involved in the selection process. Characteristics of the personalized words were also documented, including part of speech and semantic category. Terminology used to describe personalization was recorded as it appeared in the original studies, with minor normalization (e.g., singular/plural, spelling variants) for consistency. These descriptors were entered into a shared codebook and independently coded by two reviewers, with disagreements resolved through consensus. As a planned synthesis step, descriptors were subsequently organized into broader conceptual dimensions to allow for cross-study comparison. When studies included multiple participant groups or intervention types, only data pertaining to participants and treatment conditions meeting the review's inclusion criteria were extracted and synthesized. Data extraction was completed using a structured form developed for this review (see Table 1).

**Table 1.** Data extraction of study characteristics and methods of personalization.

Author (year)	Diagnosis	Pers. terms	Method of pers.	Participant involvement	Caregiver involvement	Part(s) of speech	Semantic categories
Cherney et al. (2015)	Poststroke aphasia (chronic, nonfluent *n* = 7, fluent *n* = 1)	Relevant, functional	INT, SCR	Yes	Yes	Nouns, noun phrases, verbs, verb phrases	Script-based (e.g., catching up, making plans, restaurant, cooking)
Conley & Coelho (2003)	Poststroke aphasia (chronic, Broca's)	Familiar, frequent	n.s.	Yes	n.s.	Nouns	n.s.
Croot et al. (2019)	Primary progressive aphasia (nfvPPA *n* = 3, lvPPA *n* = 2, svPPA *n* = 2, mixed *n* = 1)	Relevant, familiar	INT	Yes	Yes	Nouns, proper nouns, adjectives, verbs	n.s.
Croot et al. (2015)	Primary Progressive aphasia (nfvPPA *n* = 1, lvPPA *n* = 1)	Relevant	INT	Yes	Yes	Nouns, noun phrases, proper nouns, adjectives, adjective–noun phrases, verb participles	People, holidays, places, hobbies, life memories, household items
Fillingham et al. (2006)	Poststroke aphasia (chronic, type n.s.)	Relevant	PERF, FL	Yes	Yes	n.s.	n.s.
Flurie et al. (2020)	Primary progressive aphasia (svPPA *n* = 7, lvPPA *n* = 2)	Salient, familiar, frequent, utility	CAT	Yes	Yes	Nouns, proper nouns, verbs	Activities, clothes, hygiene, household items, places, food, people
Henry et al. (2019)	Primary progressive aphasia (svPPA *n* = 9, lvPPA *n* = 9)	Functional, relevant, important	FL, PERF	Yes	n.s.	n.s.	n.s.
Heide et al. (2023)	Poststroke aphasia (etiology: 2 ischemic, 1 arterial dissection)	Relevant	INT, CAT	Yes	n.s.	Nouns, compound nouns, verbs, adjectives	Family, grocery, kitchen items, weather, laundry, living at home, in the morning, cleaning, holidays, city life and traffic, nature and environment, COVID-19 pandemic
Hung et al. (2017)	Primary progressive aphasia (svPPA *n* = 3, lvPPA *n* = 1)	Familiar, frequent, functional, relevant	CAT, FL	Yes	Yes	Nouns, proper nouns, light verbs	People, places, food, clothes, household items, activities, personal care/hygiene
Jokel et al. (2006)	Semantic dementia	Functional, relevant	PERF, CAT	Yes	No	n.s.	Musical instruments, personal care and hygiene, household items
Kristensson et al. (2022)	Poststroke aphasia (type n.s.)	Useful	FL	Yes	Yes	Nouns, verbs	n.s.
Lavoie et al. (2019)	Poststroke aphasia (type n.s.)	Functional	INT	Yes	No	n.s.	Participant's interests (e.g. traveling, gardening, carpentry) and activities of daily living (e.g., shopping, meal preparation, personal care and hygiene)
Lavoie et al. (2020)	Primary progressive aphasia (lvPPA *n* = 3, svPPA *n* = 2)	Functional, relevant, significant, useful	FL, INT	Yes	No	Nouns	n.s.
Mason et al. (2011)	Poststroke aphasia (conduction *n* = 1, anomic *n* = 1, Broca's *n* = 1)	Functional, relevant, useful	PERF, FL	Yes	Yes	Nouns, proper nouns	Food, pottery, household items, car parts, vitamins and medications, locations, food, people (e.g., family members, work colleagues), work equipment
Paek et al. (2021)	Primary progressive aphasia (svPPA *n* = 1, nfvPPA *n* = 1)	Functional	PERF, FL	Yes	Yes	Nouns, verbs	n.s.
Palmer et al. (2019)	Poststroke aphasia (at least 4 months post)	Functional, relevant, meaningful important	INT, FL	Yes	Yes	n.s.	n.s.
Savage et al. (2013)	Semantic dementia (*n* = 4)	Relevant	INT	Yes	Yes	Nouns	Household items, kitchen items, food, outdoor tools, clothing
Savage et al. (2014)	Semantic dementia (*n* = 5)	Relevant	INT	Yes	Yes	n.s.	Everyday items (e.g., food, household appliances, clothing)
Senaha et al. (2010)	Semantic dementia (*n* = 3)	Relevant	PERF, INT	Yes	Yes	n.s.	n.s.
Sheppard et al. (2025)	Primary progressive aphasia (svPPA *n* = 3, nfvPPA *n* = 3, lvPPA *n* = 2)	Functional	PERF, INT	Yes	No	Verbs	n.s.
Zannino et al. (2025)	Poststroke aphasia (chronic, type n.s.)	Relevant	CAT	Yes	Yes	Nouns, adjectives, verbs	Kitchen items, food, household items, entertainment, children's school and games, nature and environment, health, time, numbers, festivities, clothes, accessories, descriptive terms, personal care and hygiene, travel, places, actions, people, personal items

*Note.* Coding scheme for the methods by which words were personalized are as follows: INT = interview/conversation based; SCR = script based; PERF = performance based; FL = free listing; CAT = category based. Pers. = Personalization; n.s. = not stated; nfvPPA = nonfluent variant primary progressive aphasia; lvPPA = logopenic variant primary progressive aphasia; svPPA = semantic variant primary progressive aphasia.

## Results

Figure 1 illustrates the PRISMA flowchart detailing the study selection process. The database search initially identified 1,755 records, with an additional 27 imported in via citation chain searching. Following de-duplication, 886 titles and abstracts were screened from which 40 articles were retrieved for full-text evaluation. Of those, 21 met inclusion criteria and were included in the final review.

Table 1 provides the data extracted for each of the 21 articles analyzed. In what follows, we describe the data with reference to our three research questions.

### Defining Personalized Words

For each study, we attempted to extract operational definitions of personalization used for the selection of stimulus words. In our attempts, it was clear that there were no explicit operational definitions provided; rather, studies often described the process of personalization (i.e., method) but did not provide a definition of personalization.

When examining the description of the personalization process, we identified a common set of descriptor words used across studies that infer a potential operational definition of personalization. The set of descriptor words and number of articles using each word are relevant (*n* = 15), functional (*n* = 10), familiar (*n* = 4), useful (*n* = 3), frequent (*n* = 3), important (*n* = 2), salient (*n* = 1), utility (*n* = 1), significant (*n* = 1), and meaningful (*n* = 1). Many of these terms were used while referencing prior studies that discuss the importance of personalization in treatment design (e.g., Renvall et al., 2013a, 2013b).

While there seem to be patterns in the set of descriptor terms, we opted to use a more data-driven approach to identify different sets of related descriptor terms. In doing so, we aimed to identify “dimensions” of personalization. With the set of 10 descriptor words, we obtained the semantic similarity of each pair of words using the LESK measure in WordNet (Banerjee & Pedersen, 2002); here, the relatedness of two words is determined based on the extent of overlap between the dictionary definitions of the words. For example, the words “important” and “frequent” had a relatively low LESK value of 19, while the words “important” and “significant” had a relatively high LESK value of 1,127. Next, we computed a network representation of the set of descriptor words as nodes with weighted edges based on LESK values to visualize potential dimensions of personalization (see Figure 2; all LESK values are available in Supplemental Material S1).

**Figure 2.**
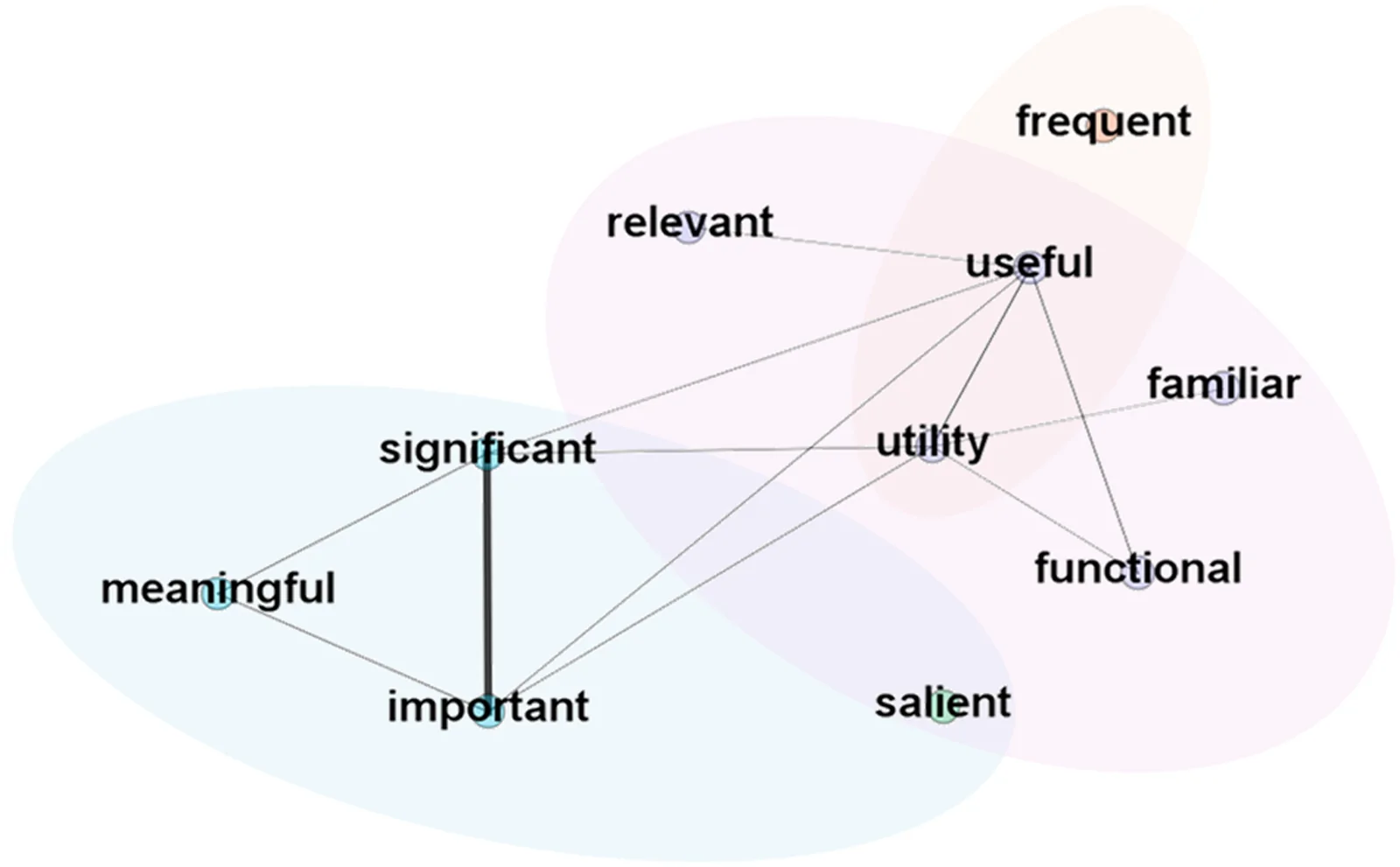
Network visualization of semantic relationships among personalization-related terms. The depicted network is filtered such that edges with a LESK value of less than 40 were removed for ease of network visualization.

Using a community detection algorithm in Gephi 0.9.7, we identified four groupings of descriptor terms, where the words were more closely connected to each other than to other words in the network. The first community consisted of the terms “meaningful,” “significant,” and “important.” The second community consisted of the terms “relevant,” “useful,” “utility,” “familiar,” and “functional.” The third community consisted of the term “salient,” which was connected also to the first and second communities. Finally, the fourth community consisted of the term “frequent,” which was also connected to the terms “useful” and “utility.”

Based on these four communities, we opted to define three dimensions that should be considered when operationalizing personalization: meaningful, functional, and frequent. Note that while the term “salience” has been frequently associated with the concept of personalization in the literature, the term “salient” is ambiguously used (Renvall et al., 2013a) and was only noted in one study of our scoping review; thus, we did not consider salience as a separate dimension.

### Methods for Selecting Personalized Words

For each study, we extracted the method of personalization and whether the participant and/or a communication partner were included in personalization approaches. After assessing the different personalization methods, we identified a common set of methods across studies: free listing (generation of words without constraints; e.g., list 100 words you want to say), interview/conversation based (words elicited through structured or semistructured conversations about needs, activities, interests, or goals), script based (words derived from co-constructed scripts; e.g., ordering food), category based (words selected from prespecified categories; e.g., food, hobbies, household items), and performance based (words chosen based on observed communication difficulty or error patterns; e.g., words named incorrectly across multiple naming attempts). As noted in the Method section above, studies only using performance-based methods were not included in the study identification process, an issue we return to later in the Discussion section. Finally, we also included a method code for “not specified.”

Regarding the general approaches used: The interview/conversation-based approach was used in 11 studies, free listing was used in eight studies, performance-based approaches were used in seven studies, the selection of words from prespecified categories was used in five studies, the script-based approach was used in one study, and one study did not clearly specify the methodological approach. Twelve of the 21 studies included more than one methodological step in personalization. All 21 studies included the involvement of participants with aphasia in personalization of word targets, while 14 studies also included the involvement of communication partners or caregivers.

### Characteristics of Personalized Words

For each study, we extracted information about the types and characteristics of words selected through the personalization process. Studies varied in the level of detail provided about the words chosen. The most consistently described characteristics were part of speech and categories.

The most common part of speech considered was nouns (*n* = 12), including noun phrases, compound nouns, and proper nouns, followed by verbs (*n* = 9), including verb phrases and specific types of verbs (participles, light verbs). A smaller number of studies also included adjectives (*n* = 3). Of the 21 studies, seven studies did not clearly specify the part of speech for selected personalized words.

Personalized words were also described in reference to different semantic categories, whether or not categories were explicitly used as a part of the methodological approach. Ten studies did not describe personalized words by category. There was a large range of categories noted in the remaining 11 studies. Categories noted in more than one study include household items (*n* = 8), people (*n* = 6), food (*n* = 6), places/locations (*n* = 5), hobbies/activities/interests (*n* = 4), clothes (*n* = 4), personal care/hygiene (*n* = 4), holidays/festivities (*n* = 3), kitchen items (*n* = 3), and nature/environment (*n* = 2). There were an additional 25 categories that were noted in a single study (Zannino et al., 2025), highlighting the idiosyncrasy in personalization of words (see Table 1).

## Discussion

The purpose of this scoping review was to examine how personalization of treatment words has been defined and implemented in anomia interventions for people with aphasia following stroke or PPA. Our review aimed to identify how prior studies define personalized words, the methods used to select them, and the linguistic characteristics of the selected words. Across the 21 included studies, personalization was frequently referenced but rarely defined explicitly. Instead, researchers described procedural approaches to personalization, most often targeting nouns and concrete categories such as household items, people, and food. These choices suggest that personalization in practice has emphasized tangible, contextually grounded vocabulary over more abstract or relational language. Through analysis of recurring descriptors, three overarching dimensions emerged, which capture how personalization has been conceptualized and implemented in the existing literature: The first dimension, *meaningful*, captures the personally valued or identity-linked aspects of therapy content, aligning with how clinicians consider what matters most to the individual with aphasia. The second dimension, *functional*, reflects practical or communicative utility, emphasizing words that support participation in daily life or are contextually accessible. The third dimension, *frequent*, captures how often the word is encountered or used in daily life. Thus, our results suggest that researchers may implicitly conceptualize personalization as the intersection of (a) what is personally meaningful, (b) what is functionally useful, and (c) what is frequently experienced or needed in communication.

While the concept of personalization is broadly valued in anomia treatment, it remains inconsistently defined and reported. Indeed, the absence of clear definitions for terms such as relevant, functional, and useful reflects a long-standing conceptual gap in aphasia rehabilitation. Recent frameworks have emphasized the importance of personalization and personal relevance in aphasia rehabilitation. Thiessen and Brown (2021) emphasize that therapy should be aligned to a patient's relative linguistic strengths and weaknesses as well as their personal goals and preferences. However, their account, like much of the broader literature, expresses why personalization matters rather than how it should be defined in practice. Over a decade ago, Renvall et al. (2013a) identified the field's tendency to refer to personalized vocabulary as “functionally relevant” without specifying what the term might denote or how the construct should be defined. Our findings indicate that this issue remains unresolved: Researchers continue to treat personalization as a guiding principle rather than a clearly operationalized construct. Across studies of anomia treatment, the same descriptors—relevant, functional, familiar, useful, and meaningful—are reiterated, but their conceptual boundaries remain largely unclarified between and within studies.

The persistent lack of definitional clarity surrounding personalization is consistent with challenges seen in adjacent fields with similarly diffuse constructs. For example, in dementia care, Tierney and Beattie (2020) conducted a concept analysis of “meaningful activity” and identified a set of fundamental attributes (e.g., enjoyment, engagement, and individualization) that capture what makes an activity meaningful in the absence of an agreed-upon definition. Their work demonstrates the value of identifying core attributes when terminology becomes overextended yet underdefined. To address this, we used a data-driven lexical analysis to examine how these descriptors relate to one another. We found that descriptors of personalization were interrelated rather than distinct, forming conceptually connected clusters. Indeed, among the dimensions used to describe personalization, the concept of “relevance” was frequently referenced, appearing in six of the nine studies (Cherney et al., 2015; Croot et al., 2015, 2019; Lavoie et al., 2020; Mason et al., 2011; Palmer et al., 2019). Other studies did not define “personally relevant” directly but referred to related constructs such as familiarity or functionality. For example, Conley and Coelho (2003) operationalized relevance in terms of “how often the individual encountered the object nouns in her everyday life” (p. 203). Studies by Croot et al. (2015, 2019) and Lavoie et al. (2019, 2020) referenced personal familiarity or daily life activities to guide word selection but did not offer formal definitions. The recurrence of these overlapping descriptors reinforces the need for a unified framework, like the three dimensions we have identified, to guide consistent reporting and implementation of personalized words in anomia treatment.

Even with inconsistent definitions of personalization, it remains a best practice recommendation to personalize therapy to a client's strengths, challenges, and experiences, as this improves engagement, satisfaction, and outcomes (Cherney et al., 2015; Simmons-Mackie et al., 2017; Thiessen & Brown, 2021). Indeed, a recent clinician survey found that word choice in treatment is driven primarily by client preference rather than by psycholinguistic or theoretical criteria (Castro & Hawes, 2025). Yet, there remains wide variation in how personalization is implemented, particularly in the methods used to identify and select treatment words (Renvall et al., 2013a). Across the studies included in this review, interview- and conversation-based approaches were used most often, followed by free listing and category-based selection methods. Interview approaches appear to be the dominant strategy for personalization, reflecting an emphasis on eliciting personalized lexical targets through direct dialogue about goals, routines, and interests. Free listing provided a more efficient but less guided alternative for gathering individualized words, whereas category-based methods represent structured attempts to balance personal relevance with a constrained stimulus set. The use of multiple methods within over half of the studies suggests that researchers and clinicians often combine strategies for personalization. Some studies paired free listing or category-based selection with performance-based assessments to further refine their words, using naming accuracy across multiple baselines to confirm which words were both personally relevant and have potential to demonstrate responsiveness to treatment. All studies involved people with aphasia in the selection process, and two thirds included communication partners or caregivers—an encouraging trend consistent with family- and participation-centered models of care, such as LPAA (e.g., Chapey et al., 2000; Rogalski & Khayum, 2018). However, the extent and nature of partner involvement varied widely, with limited reporting on how partner input shaped final stimulus selection. Rather than a single standardized framework, greater consistency and transparency are needed in how personalization is implemented and reported. Developing shared reporting guidelines would enable clearer comparison across studies while maintaining the flexibility necessary to tailor methods to each client's unique communication profile, goals, and therapy context.

Across studies, the personalized words selected for treatment were most often nouns, including proper nouns and noun phrases, with fewer studies targeting verbs and only a small number including adjectives. This pattern suggests that personalization has largely focused on concrete, imageable items that are easier to elicit, measure, and train in structured tasks, which is generally typical of anomia treatment broadly (e.g., Verb Network Strengthening Treatment; cf. Abstract Semantic Associative Network Training, which explicitly uses abstract words). Consistent with Palmer et al. (2017), words drawn from categories such as household items, people, and food were especially common, reflecting a practical emphasis on everyday communication and functional relevance. However, the predominance of nouns and concrete vocabulary may also indicate a gap in how personalization has been applied to other lexical or grammatical forms that support discourse-level communication. Renvall et al. (2013a) has observed that intervention research tends to center on nouns, with verbs receiving comparatively less attention, even though much of everyday communication relies on other types of words such as adjectives, adverbs, and pronouns. Broader inclusion of verbs, adjectives, and relational terms could extend the concept of personalization beyond object naming toward language that more closely mirrors real-life communicative demands. It is also important to note that the predominance of concrete noun targets across studies and elicitation methods should not be interpreted as reflecting patients' broader communicative priorities; rather, when individuals are asked to think about “naming” within anomia treatment contexts, they may naturally focus on visible objects and items in their immediate environment, implicitly biasing word selection toward concrete nouns rather than other word classes.

One concern is whether emphasizing personalization risks narrowing intervention targets to an individual's immediate interests, potentially excluding vocabulary that is broadly useful but not initially salient. Personalization does not need to be limited to what a person explicitly identifies as interesting; it can also encompass communicative contexts and pragmatic demands, such as shopping, ordering food, or completing activities of daily living. From this perspective, personalization reflects alignment with communicative needs and environments, not simply interests or preferences expressed at the moment personalized words are co-developed with the clinician. The dimensions identified here offer a framework for balancing personal salience with practical use.

### Limitations and Future Directions

There are important limitations to note about our study design and the results identified to drive future directions in this area. First, there was difficulty in identifying relevant articles in our scoping review. We had to conduct backward searching because studies did not consistently use the term “personally relevant” or “personalization” as we expected, and we had to exclude articles because personalization was included in reference to performance accuracy only. It is possible that there are additional studies not included in our scoping review because of the types of terms (or lack of) used in reporting personalization of treatment targets.

Second, we had intended to obtain more thorough information about the linguistic properties of selected personalized words. However, it became quickly apparent that many studies do not share stimulus sets (e.g., in appendices), likely in part due to each participant having a different set of personal words (i.e., multiple and/or long appendices would be needed). While sharing the specific words would allow for retrospective analysis of personal relevance, we were surprised by the limited description of the chosen words themselves, even in the methods section of papers. Seven studies did not even include part of speech information, and most studies did not include commonly considered psycholinguistic properties known to affect word retrieval and treatment outcomes, like lexical frequency, age of acquisition, and word length (Bemani et al., 2022; Gordon, 2002; Vonk et al., 2019). It is quite possible that these psycholinguistic properties may correlate to some degree with the dimensions of personal relevance we noted, but without the data provided, no such claims can be unambiguously made at this time. Such issues of reporting participant and treatment characteristics in aphasia treatment studies are not uncommon (e.g., RELEASE Collaboration, 2020), and our findings provide another opportunity to improve our reporting practices.

Third, the current state of personalization, based on our findings, suggests that personalization is an all-or-none aspect, either words are personalized or they are not. But our results suggest (a) that there are dimensions to personalization and (b) that some dimensions are prioritized more in some studies than in other studies. Future studies are needed to determine how these dimensions may exist on a gradient.

Fourth, it is important to note that the present review was not designed to examine the effects of personalization on anomia treatment outcomes, as such effects were not systematically evaluated across the included studies. As a result, the review could not assess whether or how personalization strategies interact with treatment intensity or total therapy duration. For example, it remains unclear whether different approaches to prioritizing personalized targets are adopted in brief interventions compared to longer or more intensive treatment programs, or whether emphasizing particular personalization dimensions is associated with differential treatment outcomes. Future research that explicitly links personalization strategies, treatment dose, and anomia outcomes will be necessary to address these questions

Finally, the present review focused specifically on anomia interventions in aphasia, reflecting the depth of this literature and the central role of lexical retrieval in aphasia rehabilitation. While this focus allowed for a detailed examination of how personalization has been defined and implemented within a well-developed intervention context, it also limits the generalizability of the proposed dimensions to other clinical populations. Future research will be needed to determine whether the dimensions of personalization identified here are similarly relevant in interventions targeting other speech and language populations.

### Clinical Implications

There are multiple implications that arose from the present study. First, there is an absence of an operational definition of personalization across studies that might have an impact on clinical implementation of personalized words. Nevertheless, we suggest that the three dimensions we identified—meaningful, functional, and frequent—be used by clinicians to operationalize personalization in their clinical practice. For example, clinicians can (a) create a set of words that hold personal significance to the client, (b) determine whether those words can be used in the client's daily life, and (c) assess whether the words are frequently used in the client's regular environment. It is possible that making these three dimensions explicit during assessment and treatment will enhance transparency and create a personalized approach that is replicable.

Clinically, these dimensions can be evaluated using complementary methods. *Meaningful* words may be identified through structured interviews or supported conversations with the individual and, when appropriate, communication partners to help identify personally valued topics. *Functional* words may be evaluated by examining whether targets support participation in specific daily activities or communicative contexts (e.g., home routines and social interactions) using tools such as activity checklists or script-based probes to help support selection. *Frequent* words may be evaluated by asking individuals or communication partners to rate how often words are encountered or needed in daily life using self-report frequency rating scales that have been applied in prior work (e.g., Vogel-Eyny, 2021). This can also be done by reviewing communication diaries or by triangulating patient and care partner reports with corpus-informed frequency lists (e.g., MRC Psycholinguistic Database; Coltheart, 1981) to assess alignment with everyday language use. Making these evaluation steps explicit going forward may improve transparency and replicability while preserving person-centered flexibility.

Second, our review also found that care partners were used in some situations to elicit information regarding personally relevant words. However, there is variability in how care partner input was used to guide the selection of the individualized stimulus sets. As Flurie et al. (2020) caution, leaving item selection solely to patients or caregivers can result in vocabularies that are overly specific or low in frequency, reducing their long-term communicative value. Therefore, we suggest that clinicians make it explicit how they involve care partners in creating different word targets and how they dealt with differences that arose between care partners' choice of words and those of people with aphasia. It is possible that different words are considered relevant for different stakeholder groups and that may impact the curated word lists.

Lastly, our review also found that there are multiple approaches to eliciting personalized vocabulary (e.g., interviews, conversations, performance based) from people with aphasia. All these methods have their strengths and drawbacks. We, therefore, suggest that clinicians can adapt a flexible, multistep approach to creating personalized vocabulary by beginning with open-ended exploration of what constitutes relevance and then refining the word lists based on the abovementioned dimensions and the treatment target.

## Conclusions

Across studies of anomia treatment, personalization has been cited as an important principle but remains inconsistently defined and implemented. The term is used broadly to indicate personal or functional relevance, yet few studies specify what qualifies a treatment target as “personalized” or how such words are identified. This review demonstrates that personalization has been approached through diverse and largely procedural methods rather than a unified conceptual model. Clarifying and operationalizing the dimensions of personalization, including what makes a word meaningful, functional, or frequent in an individual's life, will be essential for advancing reproducible research and aligning clinical practice with person-centered frameworks that emphasize participation and everyday communication.

## Data Availability Statement

This study did not generate new data. All data used in this review are available from the original publications cited within the manuscript and summarized in Table 1.

## Supplementary Material

10.1044/2026_AJSLP-25-00512SMS1 Supplemental Material S1 LESK values.

## Appendix

### Complete Search Strategies

Ovid MEDLINE(R) ALL <1946 to July 05, 2024>

1 exp Aphasia/ 13560

2 exp dementia/ 216694

3 aphasia*.mp. 21288

4 dysphasia*.mp. 1391

5 dementia*.mp. 172082

6 Logopenic.mp. 646

7 (PWA or IWA or PPA or ADRD or PPA or lvPPA or svPPA or nfvPPA).m_titl. 287

8 1 or 2 or 3 or 4 or 5 or 6 or 7 296941

9 ((Personally relevant or personal relevance or “functionally relevant” or personally chosen or Meaningful or “functional context” or contextually relevant or Individualized or Personalized) adj3 (Word* or Term* or Lexicon or lexical or target or targets or vocab* or item* or Picture* or Trained or Untrained or list* or stimuli or stimulus or communication)).mp. [mp = title, book title, abstract, original title, name of substance word, subject heading word, floating sub-heading word, key word heading word, organism supplementary concept word, protocol supplementary concept word, rare disease supplementary concept word, unique identifier, synonyms, population supplementary concept word, anatomy supplementary concept word] 5277

10 (functional Term* or functional vocab* or functional communication).mp. 1742

11 (functional adj2 (word or words)).mp. 165

12 9 or 10 or 11 7162

13 8 and 12 400

EBSCO CINAHL- 369 results on 7/8/24, 358 after limiting to academic journals

((MH “Aphasia+”) OR (MH “Dementia+”) OR (TI (aphasia* OR dysphasia* OR dementia* OR Logopenic) OR AB (aphasia* OR dysphasia* OR dementia* OR Logopenic)) OR (TI (PWA or IWA or PPA or ADRD or PPA or lvPPA or svPPA or nfvPPA)))

AND

((TI (((Personally relevant or personal relevance or “functionally relevant” or personally chosen or Meaningful or “functional context” or contextually relevant or Individualized or Personalized) N3 (Word* or Term* or Lexicon or lexical or target or targets or vocab* or item* or Picture* or Trained or Untrained or list* or stimuli or stimulus or communication))) OR AB (((Personally relevant or personal relevance or “functionally relevant” or personally chosen or Meaningful or “functional context” or contextually relevant or Individualized or Personalized) N3 (Word* or Term* or Lexicon or lexical or target or targets or vocab* or item* or Picture* or Trained or Untrained or list* or stimuli or stimulus or communication))))

OR

(TI ((functional Term* or functional vocab* or functional communication)) OR AB ((functional Term* or functional vocab* or functional communication)))

OR (TI ((functional N2 (word or words))) OR AB ((functional N2 (word or words)))))

EBSCO PsycINFO- 467 results on 7/8/24, 405 after limiting to peer-reviewed

((DE “Aphasia” OR DE “Dysphasia” OR DE “Dementia” OR DE “AIDS Dementia Complex” OR DE “Alzheimer's Disease” OR DE “Dementia with Lewy Bodies” OR DE “Frontotemporal Lobar Degeneration” OR DE “Presenile Dementia” OR DE “Pseudodementia” OR DE “Senile Dementia” OR DE “Vascular Dementia”)

OR

((TI (aphasia* OR dysphasia* OR dementia* OR Logopenic) OR AB (aphasia* OR dysphasia* OR dementia* OR Logopenic)) OR (TI (PWA or IWA or PPA or ADRD or PPA or lvPPA or svPPA or nfvPPA))))

AND

((TI (((Personally relevant or personal relevance or “functionally relevant” or personally chosen or Meaningful or “functional context” or contextually relevant or Individualized or Personalized) N3 (Word* or Term* or Lexicon or lexical or target or targets or vocab* or item* or Picture* or Trained or Untrained or list* or stimuli or stimulus or communication))) OR AB (((Personally relevant or personal relevance or “functionally relevant” or personally chosen or Meaningful or “functional context” or contextually relevant or Individualized or Personalized) N3 (Word* or Term* or Lexicon or lexical or target or targets or vocab* or item* or Picture* or Trained or Untrained or list* or stimuli or stimulus or communication))))

OR

(TI ((functional Term* or functional vocab* or functional communication)) OR AB ((functional Term* or functional vocab* or functional communication)))

OR (TI ((functional N2 (word or words))) OR AB ((functional N2 (word or words)))))

Web of Science Core Collection

1: TS = (((“Personally relevant “) NEAR/3 (Word* or Term* or Lexicon or lexical or target or targets or vocab* or item* or Picture* or Trained or Untrained or list* or stimuli or stimulus or communication))) Date Run: Mon Jul 08 2024 12:56:43 GMT-0400 (Eastern Daylight Time) Results: 131

2: TS = (((“personal relevance”) NEAR/3 (Word* or Term* or Lexicon or lexical or target or targets or vocab* or item* or Picture* or Trained or Untrained or list* or stimuli or stimulus or communication))) Date Run: Mon Jul 08 2024 13:09:50 GMT-0400 (Eastern Daylight Time) Results: 73

3: TS = (((“functionally relevant”) NEAR/3 (Word* or Term* or Lexicon or lexical or target or targets or vocab* or item* or Picture* or Trained or Untrained or list* or stimuli or stimulus or communication))) Date Run: Mon Jul 08 2024 13:10:08 GMT-0400 (Eastern Daylight Time) Results: 184

4: TS = (((“personally chosen”) NEAR/3 (Word* or Term* or Lexicon or lexical or target or targets or vocab* or item* or Picture* or Trained or Untrained or list* or stimuli or stimulus or communication))) Date Run: Mon Jul 08 2024 13:10:26 GMT-0400 (Eastern Daylight Time) Results: 5

5: TS = (((Meaningful) NEAR/3 (Word* or Term* or Lexicon or lexical or target or targets or vocab* or item* or Picture* or Trained or Untrained or list* or stimuli or stimulus or communication))) Date Run: Mon Jul 08 2024 13:10:46 GMT-0400 (Eastern Daylight Time) Results: 5926

6: TS = (((“functional context”) NEAR/3 (Word* or Term* or Lexicon or lexical or target or targets or vocab* or item* or Picture* or Trained or Untrained or list* or stimuli or stimulus or communication))) Date Run: Mon Jul 08 2024 13:11:05 GMT-0400 (Eastern Daylight Time) Results: 21

7: TS = (((“contextually relevant”) NEAR/3 (Word* or Term* or Lexicon or lexical or target or targets or vocab* or item* or Picture* or Trained or Untrained or list* or stimuli or stimulus or communication))) Date Run: Mon Jul 08 2024 13:11:22 GMT-0400 (Eastern Daylight Time) Results: 56

8: TI = (((Individualized) NEAR/3 (Word* or Term* or Lexicon or lexical or target or targets or vocab* or item* or Picture* or Trained or Untrained or list* or stimuli or stimulus or communication))) Date Run: Mon Jul 08 2024 13:12:07 GMT-0400 (Eastern Daylight Time) Results: 652

9: TS = (((Personalized) NEAR/3 (Word* or Term* or Lexicon or lexical or target or targets or vocab* or item* or Picture* or Trained or Untrained or list* or stimuli or stimulus or communication))) Date Run: Mon Jul 08 2024 13:12:24 GMT-0400 (Eastern Daylight Time) Results: 7352

10: TS = (“functional word*”) Date Run: Mon Jul 08 2024 13:16:49 GMT-0400 (Eastern Daylight Time) Results: 151

11: TS = (“functional Term*” or “functional vocab*” or “functional communication”) Date Run: Mon Jul 08 2024 13:17:43 GMT-0400 (Eastern Daylight Time) Results: 2740

12: #11 OR #10 OR #9 OR #8 OR #7 OR #6 OR #5 OR #4 OR #3 OR #2 OR #1 Date Run: Mon Jul 08 2024 13:18:34 GMT-0400 (Eastern Daylight Time) Results: 17209

13: TS = (aphasia* OR dysphasia* OR dementia* OR Logopenic) Date Run: Mon Jul 08 2024 13:19:29 GMT-0400 (Eastern Daylight Time) Results: 250360

14: TI = (PWA or IWA or PPA or ADRD or PPA or lvPPA or svPPA or nfvPPA) Date Run: Mon Jul 08 2024 13:20:12 GMT-0400 (Eastern Daylight Time) Results: 1202

15: #14 OR #13 Date Run: Mon Jul 08 2024 13:20:28 GMT-0400 (Eastern Daylight Time) Results: 251403

16: #15 AND #12 Date Run: Mon Jul 08 2024 13:20:48 GMT-0400 (Eastern Daylight Time) Results: 635

17: #15 AND #12 and Article or Review Article or Early Access (Document Types) Date Run: Mon Jul 08 2024 13:29:19 GMT-0400 (Eastern Daylight Time) Results: 605

18: #15 AND #12 and Article or Review Article or Early Access (Document Types) and Construction Building Technology or Biochemistry Molecular Biology or Computer Science Cybernetics or Art or Computer Science Hardware Architecture or Computer Science Software Engineering or Economics or Hematology or History Philosophy Of Science or Mathematical Computational Biology or Philosophy (Exclude – Web of Science Categories) Date Run: Mon Jul 08 2024 13:30:26 GMT-0400 (Eastern Daylight Time) Results: 592

## References

[bib59] Banerjee, S., & Pedersen, T. (2002). An adapted Lesk algorithm for word sense disambiguation using WordNet. In A. Gelbukh (Ed.), Computational linguistics and intelligent text processing (pp. 136–145). Springer. 10.1007/3-540-45715-1_11

[bib1] Bemani, Z., Moayedfar, S., & Ghasisin, L. (2022). Psycholinguistic variables influencing word retrieval in Persian-speaking people with aphasia. Aphasiology, 36(7), 868–882. 10.1080/02687038.2021.1907292

[bib2] Brady, M. C., Kelly, H., Godwin, J., & Enderby, P. (2012). Speech and language therapy for aphasia following stroke. The Cochrane Database of Systematic Reviews, 5, Article CD000425. 10.1002/14651858.CD000425.pub3 22592672

[bib3] Cadório, I., Lousada, M., Martins, P., & Figueiredo, D. (2017). Generalization and maintenance of treatment gains in primary progressive aphasia (PPA): A systematic review. International Journal of Language & Communication Disorders, 52(5), 543–560. 10.1111/1460-6984.12310 28120406

[bib4] Castro, N., & Hawes, O. K. (2025). Factors impacting clinician selection of words used in anomia treatment. Aphasiology, 39(8), 1029–1038. 10.1080/02687038.2024.2406461

[bib5] Chapey, R., Duchan, J. F., Elman, R. J., Garcia, L. J., Kagan, A., Lyon, J. G., & Simmons Mackie, N. (2000). Life Participation Approach to Aphasia: A statement of values for the future. The ASHA Leader Archive, 5(3), 4–6. 10.1044/leader.FTR.05032000.4

[bib6] Cherney, L. R., Kaye, R. C., Lee, J. B., & van Vuuren, S. (2015). Impact of personal relevance on acquisition and generalization of script training for aphasia: A preliminary analysis. American Journal of Speech-Language Pathology, 24(4), S913–S922. 10.1044/2015_AJSLP-14-0162 PMC4698473 26340806

[bib7] Coltheart, M. (1981). The MRC psycholinguistic database. The Quarterly Journal of Experimental Psychology Section A, 33A(4), 497–505. 10.1080/14640748108400805

[bib8] Conley, A., & Coelho, C. (2003). Treatment of word retrieval impairment in chronic Broca's aphasia. Aphasiology, 17(3), 203–211. 10.1080/729255460

[bib9] Cordella, C., Di Filippo, L., Kolachalama, V. B., & Kiran, S. (2024). Connected speech fluency in poststroke and progressive aphasia: A scoping review of quantitative approaches and features. American Journal of Speech-Language Pathology, 33(4), 2083–2120. 10.1044/2024_AJSLP-23-00208 PMC11253646 38652820

[bib11] Croot, K., Raiser, T., Taylor-Rubin, C., Ruggero, L., Ackl, N., Wlasich, E., Danek, A., Scharfenberg, A., Foxe, D., Hodges, J. R., Piguet, O., Kochan, N. A., & Nickels, L. (2019). Lexical retrieval treatment in primary progressive aphasia: An investigation of treatment duration in a heterogeneous case series. Cortex, 115, 133–158. 10.1016/j.cortex.2019.01.009 30822613

[bib12] Croot, K., Taylor, C., Abel, S., Jones, K., Krein, L., Hameister, I., Ruggero, L., & Nickels, L. (2015). Measuring gains in connected speech following treatment for word retrieval: A study with two participants with primary progressive aphasia. Aphasiology, 29(11), 1265–1288. 10.1080/02687038.2014.975181

[bib13] Dietz, A., Weissling, K., Griffith, J., & McKelvey, M. (2013). Personalizing AAC for people with aphasia: The role of text and pictures [Paper presentation]. 43rd Clinical Aphasiology Conference, Tucson, AZ. https://digitalcommons.unl.edu/specedfacpub/154

[bib14] Fillingham, J., Sage, K., & Ralph, M. (2006). The treatment of anomia using errorless learning. Neuropsychological Rehabilitation, 16(2), 129–154. 10.1080/09602010443000254 16565031

[bib15] Flurie, M., Ungrady, M., & Reilly, J. (2020). Evaluating a maintenance-based treatment approach to preventing lexical dropout in progressive anomia. Journal of Speech, Language, and Hearing Research, 63(12), 4082–4095. 10.1044/2020_JSLHR-20-00059 PMC8608146 33181044

[bib16] Fridriksson, J., & Hillis, A. E. (2021). Current approaches to the treatment of poststroke aphasia. Journal of Stroke, 23(2), 183–201. 10.5853/jos.2020.05015 PMC8189855 34102754

[bib17] Gordon, J. K. (2002). Phonological neighborhood effects in aphasic speech errors: Spontaneous and structured contexts. Brain and Language, 82(2), 113–145. 10.1016/s0093-934x(02)00001-9 12096871

[bib18] Heide, J., Netzebandt, J., Ahrens, S., Brüsch, J., Saalfrank, T., & Schmitz-Antonischki, D. (2023). Improving lexical retrieval with LingoTalk: An app-based, self-administered treatment for clients with aphasia. Frontiers in Communication, 8, Article 1210193. 10.3389/fcomm.2023.1210193

[bib19] Henry, M. L., Hubbard, H. I., Grasso, S. M., Dial, H. R., Beeson, P. M., Miller, B. L., & Gorno-Tempini, M. L. (2019). Treatment for word retrieval in semantic and logopenic variants of primary progressive aphasia: Immediate and long-term outcomes. Journal of Speech, Language, and Hearing Research, 62(8), 2723–2749. 10.1044/2018_JSLHR-L-18-0144 PMC6802912 31390290

[bib20] Hung, J., Bauer, A., Grossman, M., Hamilton, R. H., Coslett, H. B., & Reilly, J. (2017). Semantic feature training in combination with transcranial direct current stimulation (tDCS) for progressive anomia. Frontiers in Human Neuroscience, 11, Article 253. 10.3389/fnhum.2017.00253 PMC5432627 28559805

[bib22] Jokel, R., Graham, N. L., Rochon, E., & Leonard, C. (2014). Word retrieval therapies in primary progressive aphasia. Aphasiology, 28(8–9), 1038–1068. 10.1080/02687038.2014.899306

[bib23] Jokel, R., Rochon, E., & Leonard, C. (2006). Treating anomia in semantic dementia: Improvement, maintenance, or both? Neuropsychological Rehabilitation, 16(3), 241–256. 10.1080/09602010500176757 16835150

[bib24] Kleim, J. A., & Jones, T. A. (2008). Principles of experience-dependent neural plasticity: Implications for rehabilitation after brain damage. Journal of Speech, Language, and Hearing Research, 51(1), S225–S239. 10.1044/1092-4388(2008/018) 18230848

[bib25] Kristensson, J., Saldert, C., Östberg, P., Smith, S., Åke, S., & Longoni, F. (2022). Naming vs. non-naming treatment in aphasia in a group setting—A randomized controlled trial. Journal of Communication Disorders, 97, Article 106215. 10.1016/j.jcomdis.2022.106215 35367876

[bib26] Larfeuil, C., & Dorze, G. L. (1997). An analysis of the word-finding difficulties and of the content of the content of the discourse of recent and chronic aphasic speakers. Aphasiology, 11(8), 783–811. 10.1080/02687039708250456

[bib27] Lavoie, M., Bier, N., Laforce, R., & Macoir, J. (2020). Improvement in functional vocabulary and generalization to conversation following a self-administered treatment using a smart tablet in primary progressive aphasia. Neuropsychological Rehabilitation, 30(7), 1224–1254. 10.1080/09602011.2019.1570943 30714482

[bib28] Lavoie, M., Bier, N., & Macoir, J. (2019). Efficacy of a self-administered treatment using a smart tablet to improve functional vocabulary in poststroke aphasia: A case-series study. International Journal of Language & Communication Disorders, 54(2), 249–264. 10.1111/1460-6984.12439 30426650

[bib29] Mason, C., Nickels, L., McDonald, B., Moses, M., Makin, K., & Taylor, C. (2011). Treatment of word retrieval impairments in aphasia: Evaluation of a self-administered home programme using personally chosen words. Aphasiology, 25(2), 245–268. 10.1080/02687038.2010.489258

[bib30] Mayer, J. F., Madden, E. B., Mozeiko, J., Murray, L. L., Patterson, J. P., Purdy, M., Sandberg, C. W., & Wallace, S. E. (2024). Generalization in aphasia treatment: A tutorial for speech-language pathologists. American Journal of Speech-Language Pathology, 33(1), 57–73. 10.1044/2023_AJSLP-23-00192 38052053

[bib31] National Institutes of Health. (n.d.). Primary progressive aphasia [Genetic and Rare Diseases Information Center]. https://rarediseases.info.nih.gov/diseases/8541/primary-progressive-aphasia

[bib32] Nickels, L. (2002). Therapy for naming disorders: Revisiting, revising, and reviewing. Aphasiology, 16(10–11), 935–979. 10.1080/02687030244000563

[bib33] Paek, E., Murray, L., & Newman, S. (2021). Effects of concurrent action and object naming treatment on naming skills and functional brain activation patterns in primary progressive aphasia: An fMRI study with a case-series design. Brain and Language, 218, Article 104950. 10.1016/j.bandl.2021.104950 33836414

[bib34] Palmer, R., Dimairo, M., Cooper, C., Enderby, P., Brady, M., Bowen, A., Latimer, N., Julious, S., Cross, E., Alshreef, A., Harrison, M., Bradley, E., Witts, H., & Chater, T. (2019). Self-managed, computerised speech and language therapy for patients with chronic aphasia poststroke compared with usual care or attention control (Big CACTUS): A multicentre, single-blinded, randomised controlled trial. The Lancet Neurology, 18(9), 821–833. 10.1016/S1474-4422(19)30192-9 PMC6700375 31397288

[bib35] Palmer, R., Hughes, H., & Chater, T. (2017). What do people with aphasia want to be able to say? A content analysis of words identified as personally relevant by people with aphasia. PLOS ONE, 12(3), Article e0174065. 10.1371/journal.pone.0174065 PMC5367780 28346518

[bib36] Peters, M. D. J., Marnie, C., Tricco, A. C., Pollock, D., Munn, Z., Alexander, L., McInerney, P., Godfrey, C. M., & Khalil, H. (2020). Updated methodological guidance for the conduct of scoping reviews. JBI Evidence Synthesis, 18(10), 2119–2126. 10.11124/JBIES-20-00167 33038124

[bib37] Raymer, A. M., Beeson, P., Holland, A., Kendall, D., Maher, L. M., Martin, N., Murray, L., Rose, M., Thompson, C. K., Turkstra, L., Altmann, L., Boyle, M., Conway, T., Hula, W., Kearns, K., Rapp, B., Simmons-Mackie, N., & Gonzalez Rothi, L. J. (2008). Translational research in aphasia: From neuroscience to neurorehabilitation. Journal of Speech, Language, and Hearing Research, 51(1), S259–S275. 10.1044/1092-4388(2008/020) 18230850

[bib38] Raymer, A. M., & Roitsch, J. (2023). Word retrieval treatments in aphasia: A survey of professional practice. Aphasiology, 37(7), 954–979. 10.1080/02687038.2022.2063791

[bib39] Reilly, J., Peelle, J. E., Antonucci, S. M., & Grossman, M. (2011). Anomia as a marker of distinct semantic memory impairments in Alzheimer's disease and semantic dementia. Neuropsychology, 25(4), 413–426. 10.1037/a0022738 PMC3125450 21443339

[bib40] RELEASE Collaboration. (2020). Communicating simply, but not too simply: Reporting of participants and speech and language interventions for aphasia after stroke. International Journal of Speech-Language Pathology, 22(3), 302–312. 10.1080/17549507.2020.1762000 32686592

[bib41] Renvall, K., Nickels, L., & Davidson, B. (2013a). Functionally relevant items in the treatment of aphasia (Part I): Challenges for current practice. Aphasiology, 27(6), 636–650. 10.1080/02687038.2013.786804

[bib42] Renvall, K., Nickels, L., & Davidson, B. (2013b). Functionally relevant items in the treatment of aphasia (Part II): Further perspectives and specific tools. Aphasiology, 27(6), 651–677. 10.1080/02687038.2013.796507

[bib43] Robinaugh, G., & Henry, M. L. (2022). Chapter 14—Behavioral interventions for primary progressive aphasia. In A. E. Hillis & J. Fridriksson (Eds.), Handbook of clinical neurology (Vol. 185, pp. 221–240). Elsevier. 10.1016/B978-0-12-823384-9.00011-6 35078600

[bib44] Rogalski, E. J., & Khayum, B. (2018). A life participation approach to primary progressive aphasia intervention. Seminars in Speech and Language, 39(3), 284–296. 10.1055/s-0038-1660786 PMC6350508 29933494

[bib45] Savage, S., Ballard, K., Piguet, O., & Hodges, J. (2013). Bringing words back to mind—Improving word production in semantic dementia. Cortex, 49(7), 1823–1832. 10.1016/j.cortex.2012.09.014 23127420

[bib46] Savage, S., Piguet, O., & Hodges, J. (2014). Giving words new life: Generalization of word retraining outcomes in semantic dementia. Journal of Alzheimers Disease, 40(2), 309–317. 10.3233/JAD-131826 24413622

[bib47] Senaha, M. L. H., Brucki, S. M. D., & Nitrini, R. (2010). Rehabilitation in semantic dementia: Study of effectiveness of lexical reacquisition in three patients. Dementia & Neuropsychologia, 4(4), 306–312. 10.1590/S1980-57642010DN40400009 PMC5619064 29213703

[bib48] Sheppard, S., Goldberg, E., Sebastian, R., Vitti, E., Ruch, K., Meier, E., & Hillis, A. (2025). Augmenting verb-naming therapy with neuromodulation decelerates language loss in primary progressive aphasia. American Journal of Speech-Language Pathology, 34(1), 155–173. 10.1044/2024_AJSLP-24-00016 PMC11745310 39666609

[bib49] Simmons-Mackie, N., & Cherney, L. R. (2018). Aphasia in North America: Highlights of a white paper. Archives of Physical Medicine and Rehabilitation, 99(10), Article e117. 10.1016/j.apmr.2018.07.417

[bib50] Simmons-Mackie, N., Worrall, L., Murray, L. L., Enderby, P., Rose, M. L., Paek, E. J., & Klippi, A. (2017). The top ten: Best practice recommendations for aphasia. Aphasiology, 31(2), 131–151. 10.1080/02687038.2016.1180662

[bib51] Thiessen, A., & Brown, J. (2021). Personalization of restorative and compensatory treatments for people with aphasia: A review of the evidence. Topics in Language Disorders, 41(3), Article 269. 10.1097/TLD.0000000000000253

[bib52] Tierney, L., & Beattie, E. (2020). Enjoyable, engaging and individualised: A concept analysis of meaningful activity for older adults with dementia. International Journal of Older People Nursing, 15(2), Article e12306. 10.1111/opn.12306 32022414

[bib53] Tricco, A. C., Lillie, E., Zarin, W., O'Brien, K. K., Colquhoun, H., Levac, D., Moher, D., Peters, M. D. J., Horsley, T., Weeks, L., Hempel, S., Akl, E. A., Chang, C., McGowan, J., Stewart, L., Hartling, L., Aldcroft, A., Wilson, M. G., Garritty, C., … Straus, S. E. (2018). PRISMA Extension for Scoping Reviews (PRISMA-ScR): Checklist and explanation. Annals of Internal Medicine, 169(7), 467–473. 10.7326/M18-0850 30178033

[bib54] Van Stan, J. H., Dijkers, M. P., Whyte, J., Hart, T., Turkstra, L. S., Zanca, J., & Chen, C. (2019). The Rehabilitation Treatment Specification System: Implications for improvements in research design, reporting, replication, and synthesis. Archives of Physical Medicine and Rehabilitation, 100(1), 146–155. 10.1016/j.apmr.2018.09.112 PMC6452635 30267666

[bib10] Veritas Health Innovation. (n.d.). Covidence system review software [Computer software]. www.covidence.org

[bib55] Vogel-Eyny, A. (2021). Predictors of lexical accessibility of common and proper nouns in older age: Evidence from the tip-of-the-tongue state [Doctoral dissertation, City University of New York]. CUNY Academic Works. https://academicworks.cuny.edu/gc_etds/4367

[bib56] Vonk, J. M. J., Jonkers, R., Hubbard, H. I., Gorno-Tempini, M. L., Brickman, A. M., & Obler, L. K. (2019). Semantic and lexical features of words dissimilarly affected by non-fluent, logopenic, and semantic primary progressive aphasia. Journal of the International Neuropsychological Society, 25(10), 1011–1022. 10.1017/S1355617719000948 PMC6879829 31511121

[bib57] Webster, J., Whitworth, A., & Morris, J. (2015). Is it time to stop “fishing”? A review of generalisation following aphasia intervention. Aphasiology, 29(11), 1240–1264. 10.1080/02687038.2015.1027169

[bib21] World Health Organization. (n.d.). International Classification of Functioning, Disability and Health (ICF). Retrieved October 22, 2025, from https://www.who.int/standards/classifications/international-classification-of-functioning-disability-and-health

[bib58] Zannino, G., Stanzione, A., Di Laurenzio, R., Annicchiarico, R., Formisano, R., Caltagirone, C., & Carlesimo, G. (2025). A self-administered fully automated protocol for remediating word finding difficulties in chronic aphasic persons: Preliminary data. Aphasiology. Advance online publication. 10.1080/02687038.2025.2514532

