# Supplementary material for: Personalization of Words in Anomia Treatment for People With Aphasia: A Scoping Review
Source: Am J Speech Lang Pathol. 2026 May 8;35(5 Suppl):2867–82. doi: 10.1044/2026_AJSLP-25-00512 (PMC13684822; doi:10.1044/2026_AJSLP-25-00512)
Supplement: Supplementary file 1 [file AJSLP-35-2867-s001.pdf]

Supplemental Material S1. LESK values.

	Relevant	Functional	Familiar	Useful	Frequent	Salient	Utility	Significant	Meaningful
Relevant									
Functional	19								
Familiar	16	22							
Useful	45	133	33						
Frequent	12	17	15	21					
Salient	19	16	15	23	10				
Utility	28	59	41	174	28	33			
Significant	30	34	35	75	19	22	78		
Meaningful	22	27	21	37	15	15	31	107	
Important	33	34	35	85	19	28	88	1127	107
